# Exploring Neuroprotection against Radiation-Induced Brain Injury: A Review of Key Compounds

**DOI:** 10.3390/neurosci5040034

**Published:** 2024-10-12

**Authors:** Lucas González-Johnson, Ariel Fariña, Gonzalo Farías, Gustavo Zomosa, Víctor Pinilla-González, Catalina Rojas-Solé

**Affiliations:** 1 Faculty of Medicine, Universidad de Chile, Santiago 8330111, Chile; gofarias@uchile.cl (G.F.); victorpinilla@ug.uchile.cl (V.P.-G.); catalinarojass@ug.uchile.cl (C.R.-S.); 2 University of Chile Clinical Hospital, Santiago 8380453, Chile; gzomosar@hotmail.com; 3 Biomedical Neuroscience Institute (BNI), Faculty of Medicine, Universidad de Chile, Santiago 8330111, Chile; 4 Fundación Arturo López Pérez, Santiago 7500921, Chile; dr.ariel.farina@gmail.com; 5 Faculty of Medicine, Universidad de los Andes, Santiago 12455, Chile; 6 Molecular and Clinical Pharmacology Program, Institute of Biomedical Sciences, Faculty of Medicine, University of Chile, Santiago 8330111, Chile

**Keywords:** cognitive impairment, radiation-induced brain injury, free radicals, translational medicine, whole brain radiation therapy, ascorbate

## Abstract

Brain radiation is a crucial tool in neuro-oncology for enhancing local tumor control, but it can lead to mild-to-profound and progressive impairments in cognitive function. Radiation-induced brain injury is a significant adverse effect of radiotherapy for cranioencephalic tumors, primarily caused by indirect cellular damage through the formation of free radicals. This results in late neurotoxicity manifesting as cognitive impairment due to free radical production. The aim of this review is to highlight the role of different substances, such as drugs used in the clinical setting and antioxidants such as ascorbate, in reducing the neurotoxicity associated with radiation-induced brain injury. Currently, there is mainly preclinical and clinical evidence supporting the benefit of these interventions, representing a cost-effective and straightforward neuroprotective strategy.

## 1. Introduction

Radiotherapy (RT) is commonly used to treat various central nervous system (CNS) tumors, including glioblastomas, germinomas, vestibular schwannomas, and brain metastases (BMs) [1]. It is estimated that 20–50% of patients with malignant neoplasms will develop brain metastases during the course of their disease [2]. In this context, radiosurgery and Whole Brain Radiation Therapy (WBRT) play a crucial role, becoming key therapeutic tools for managing both primary and secondary brain tumors [3]. These techniques are particularly useful for treating tumors located in encephalic regions that are difficult to access surgically and as an adjuvant therapy [4,5]. The standard WBRT regimen for BM consists of 30 Gy administered in 10 fractions (10 × 3 Gy) over 2 weeks in most centers globally [6], while radiosurgery can be delivered in a single session or in 3 to 5 fractions [5].

Both brain tumors and their treatments can result in neurocognitive impairment [7]. Traditional neuro-oncological perspectives tend to oversimplify the brain as a series of functional units organized in parallel circuits, often overlooking the significance of CNS volume in radiation dose tolerance. While small volumes of brain tissue can tolerate high radiation doses with minimal functional impact, WBRT, which affects larger volumes, is more likely to cause neurocognitive deficits [3,8]. This is primarily due to damage to normal brain parenchyma, particularly in the hippocampus, where neural stem cells reside [9]. Several randomized controlled trials have demonstrated cognitive impairment in patients following WBRT [10,11,12].

One strategy to reduce the risk of cognitive decline is hippocampal-sparing WBRT, a technique that combines intensity-modulated radiation therapy (IMRT) with hippocampal avoidance. This approach has been shown to lower the risk of cognitive impairment from 30% to 7% [13]. Another method is radiosurgery, which targets metastases with high doses of radiation while sparing normal brain tissue, maintaining effective tumor control without compromising overall survival [14]. However, these advanced techniques are available only in centers equipped with modern radiotherapy technology, leaving many patients without access to hippocampal-sparing WBRT or radiosurgery.

In this context, radiation-induced brain injury (RIBI) remains a significant adverse effect of radiotherapy for cranial tumors, with limited options available for its prevention [15]. Over the past decade, various pharmacological agents have been investigated to mitigate the cognitive toxicity associated with radiotherapy, but their results have been largely suboptimal, partly due to an incomplete understanding of the underlying mechanisms [16,17]. Recent evidence highlights oxidative stress as a key mediator of RIBI, as both in vitro and in vivo models have demonstrated that radiotherapy generates free radicals (FRs), causing indirect cellular damage through sublethal injury and promoting neuronal apoptosis [16,18]. This has spurred interest in antioxidant agents as potential therapies to alleviate RIBI. In light of this, the present manuscript reviews the role of oxidative stress in radiation-induced brain injury and explores various pharmacological agents with antioxidant properties that have been studied as radioprotectants, with a particular emphasis on ascorbic acid (AA), one of the most well-researched compounds in this field.

## 2. Principles of Radiotherapy in Normal and Cancerous Cells

The response of both tumor and normal cells to multiple doses of radiation is governed by five key factors, commonly referred to as the 5 Rs of radiobiology: (1) DNA repair, (2) redistribution in the cell cycle, (3) reoxygenation, (4) repopulation, and (5) radiosensitivity [19]. These factors are essential in optimizing radiotherapy (RT) treatments.

A critical concept in radiotherapy is the α/β ratio, a metric that describes the sensitivity of tumors and normal tissues to fractionation [1,20]. Tissue response to radiation and fractionated dosing is modeled using various frameworks, with the Linear Quadratic (LQ) model being the most widely applied. This model predicts the biological response of tissues by calculating the surviving fraction of cells after a given dose. The LQ equation incorporates two key parameters: a linear dose coefficient (α), which is more relevant at low doses, and a quadratic dose coefficient (β), which becomes significant at higher doses, typically in the 1–8 Gy range. Clinically, this model distinguishes between early-responding tissues, which have a high α/β ratio, and late-responding tissues, which have a low α/β ratio. Malignant tumors generally exhibit high α/β ratios, while slow-growing benign tumors tend to have lower ratios [21].

For late-responding normal tissues like the brain and spinal cord, the α/β ratio is typically 2–3 Gy, reflecting the low regenerative capacity of the central nervous system (CNS). In contrast, early-responding tissues such as the skin or gastrointestinal tract, along with most squamous cell carcinomas, exhibit an α/β ratio of around 10 Gy [22].

In this context, this section will present the main effects of radiation on the cells of our organism, with emphasis on the mechanisms of damage to the genetic material and its repair, as well as the main mechanisms of cell death involved.

### 2.1. Radiation-Induced DNA Damage

**Figure 1:**
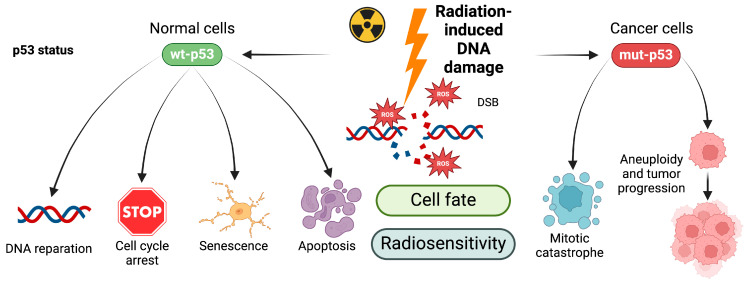
Cellular response to radiation-induced DNA damage. Ionizing radiation induces DNA damage in cells in the form of single- or double-strand breaks via ROS formation, thus blocking their ability to divide and proliferate further. DNA damage is sensed by cells, and results in various cellular responses, depending on the level of DNA damage (repairable or irreparable) and cell type (normal or cancer cell). Failure to activate normal p53-dependent DNA damage response may cause mitotic catastrophe or the generation of aneuploid cells which contribute to the progression of cancer. Signaling pathways that promote DNA repair and inhibition of cell death can protect cancer cells from irradiation-induced cytotoxicity, promoting survival and subsequent radiation resistance of cancer cells.

Radiation causes DNA damage primarily in the form of single-strand breaks (SSBs) and double-strand breaks (DSBs). These lesions are recognized by sensor proteins that activate downstream signaling pathways to initiate the DNA damage response (DDR) [23,24]. The p53 protein plays a central role in DDR, with its concentration and phosphorylation status determining whether the cell will survive or undergo apoptosis [25]. Additionally, cyclin-dependent kinase inhibitors and checkpoint kinases are involved in cell cycle arrest, allowing for DNA repair and the maintenance of genomic stability [25,26,27,28] (Figure 1).

### 2.2. Double-Strand Break Repair Mechanisms (DSB)

Double-strand breaks (DSBs) can be repaired through two primary mechanisms: non-homologous end-joining (NHEJ) and homologous recombination (HR) [27]. NHEJ, although error-prone, is active throughout the entire cell cycle, particularly in the G1 phase. Its inaccuracy stems from the processing of DNA ends before ligation, which can result in short insertions or deletions, potentially leading to the loss of genetic information or misrepair by ligating ends from different DSBs, resulting in translocations and rearrangements that may cause aneuploidy [27].

In contrast, HR is an error-free process but requires an intact sister chromatid as a repair template, making it only functional during the S and G2 phases of the cell cycle. When cellular damage exceeds repair capacity, cell death (CD) ensues [27].

The choice between NHEJ and HR for DNA repair is regulated by the competition between DNA-end protection and resection, primarily governed by BRCA1 and the X-ray repair cross-complementing proteins. Normal cells possess redundancy in DNA repair pathways, enhancing their repair capacity, whereas cancer cells often exhibit impaired DNA repair mechanisms, leading to a higher occurrence of DNA breaks. If DNA damage is successfully repaired, the cell proceeds with its cycle; otherwise, cell death is triggered [27].

### 2.3. Cell Death Mechanisms

Radiation-induced cell death (CD) as a result of impaired DNA repair depends on several factors, including cell type, TP53 status, oxygen supply, DNA repair capacity, the stage of the cell cycle during irradiation, microenvironment characteristics, radiation dose, and quality [27,29]. In most solid tumors, mitotic CD is the predominant form of cell death, primarily through mitotic catastrophe, with apoptosis playing a secondary role. In contrast, normal tissues typically undergo senescence (Figure 1).

#### 2.3.1. Apoptosis

Apoptosis is a tightly regulated form of CD characterized by pyknosis, cell shrinkage, and internucleosomal DNA fragmentation [30]. There are three main pathways leading to apoptosis: (1) intrinsic/mitochondrial, (2) extrinsic, and (3) membrane stress/ceramide pathway [27]. Apoptosis has a limited role in the response of most solid tumors to treatment, due to the loss of pro-apoptotic mechanisms during oncogenesis [31,32]. Radiation predominantly activates the intrinsic apoptotic pathway (IAP).

The IAP is initiated by DNA damage, such as single-strand breaks (SSBs) and double-strand breaks (DSBs). When DNA repair fails, prolonged activation of p53 increases the likelihood of apoptosis over cell cycle arrest by disrupting the balance between pro- and anti-apoptotic factors. This imbalance leads to the release of cytochrome c from mitochondria, activating caspase 9 and forming the apoptosome [31]. The extrinsic apoptotic pathway is initiated by external signals, such as tumor necrosis factor (TNF) ligands binding to death receptors on the plasma membrane, which activates caspase 8 [33,34]. Radiation can upregulate death receptors, making cells more susceptible to extrinsic apoptosis. The ceramide pathway, independent of DNA damage and p53, is activated by oxidative stress and membrane damage, leading to ceramide production, which acts as a second messenger in apoptosis signaling [35].

All three pathways ultimately converge on the activation of effector caspases (caspases 3 and 7), initiating the demolition phase of apoptosis, which results in controlled degradation of cellular components [35].

#### 2.3.2. Mitotic Catastrophe (MC)

Mitotic catastrophe is the primary form of p53-independent CD induced by ionizing radiation, particularly in apoptosis-resistant cells [31]. It refers to cell death occurring during, or as a result of, aberrant mitosis [36]. This process is triggered by the premature induction of mitosis before the completion of the S and G2 phases, leading to cell cycle arrest and subsequent regulated death, either during the first mitotic division (mitotic death) or in subsequent divisions via delayed apoptosis or necrosis [31,37]. This delayed response explains the delayed cell death often observed in solid tumors after RT, occurring 2–6 days post-irradiation [38]. Aberrant mitoses and failed cytokinesis result in atypical chromosome segregation and division, producing tetraploid giant cells, aneuploidy, micronuclei formation, and centrosome hyper-amplification, particularly in cells lacking functional p53 [39,40,41]. These cells become incapable of further replication [37,42,43,44].

#### 2.3.3. Senescence

Senescence refers to permanent cell cycle arrest, characterized microscopically by enlarged, flattened cells with increased granularity [45,46]. Radiation-induced senescence is triggered by DNA damage and activation of functional p53 and pRb. Although senescent cells are clonogenically “dead,” they remain metabolically active and viable for extended periods (dormant), secreting factors that can alter the tumor microenvironment (TME) and potentially promote tumor growth and progression [47,48]. In some tumors, senescence may be a mechanism to escape radiation-induced cytotoxicity, and senescent cells can potentially “reawaken” after months or years due to external stimuli in the TME [31]. Other forms of radiation-induced CD include necrosis and autophagy.

## 3. Oxidative Stress and Antioxidant Defenses in the CNS: Implications for Radiation-Induced Brain Injury

Redox reactions generate pro-oxidant reactive species that, at appropriate concentrations, are essential for various cellular and organismal functions, including defense against microorganisms, intracellular communication, and transcription factor activation. However, when these species exceed optimal levels, they can cause cellular damage, which antioxidant mechanisms strive to counteract [49,50]. The balance between oxidants and antioxidants is critical for maintaining cellular function, but various conditions can disrupt this redox homeostasis, resulting in oxidative stress, where pro-oxidants overwhelm the body’s antioxidant defenses [50].

At the cellular level, reactive oxygen species (ROS) and reactive nitrogen species (RNS) are the principal pro-oxidants, and their effects are modulated by both enzymatic and non-enzymatic antioxidant systems. Key antioxidant enzymes include superoxide dismutase (SOD), catalase (CAT), glutathione peroxidase (GPX), and thioredoxin (TRx), among others [51]. Oxidative stress induces cellular damage through lipid peroxidation, protein oxidation, and DNA strand breaks, with tissues such as the central nervous system (CNS) and muscle, which have low cell turnover, being particularly vulnerable [52,53,54].

The CNS is especially susceptible to oxidative damage, for several reasons. Firstly, it consumes approximately 20% of the body’s oxygen, which leads to excessive ROS production under pathological conditions [55,56]. Moreover, ROS are involved in critical CNS processes, such as neuronal plasticity, axonal regeneration, and neurotransmission [57]. Despite these essential roles, the CNS has limited antioxidant defenses, with relatively low levels of glutathione and catalase compared to other tissues [58,59,60]. Additionally, the abundance of redox-active transition metals and the generation of hydrogen peroxide through monoaminergic metabolism exacerbate oxidative imbalance [61,62]. The high concentration of polyunsaturated fatty acids (PUFAs) in CNS lipid membranes further increases susceptibility to lipid peroxidation and ferroptosis [63,64].

**Figure 2:**
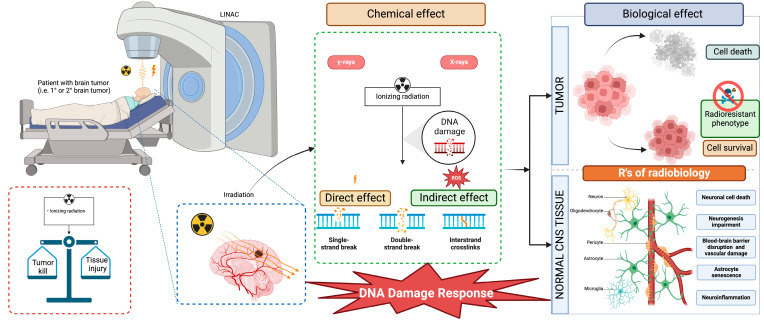
Principles of radiotherapy in normal and cancerous cells. This figure illustrates the distinct responses of normal and tumor cells to radiation. It emphasizes the pathways through which radiation induces DNA damage, ultimately leading to various forms of cell death, including apoptosis, mitotic catastrophe, and senescence. Abbreviations: CNS, Central Nervous System; ROS, Reactive Oxygen Species.

Oxidative stress also plays a critical role in the pathogenesis of radiation-induced brain injury (RIBI). Ionizing radiation generates substantial amounts of ROS and free radicals through the radiolysis of water, leading to extensive cellular damage, particularly to DNA [65] (Figure 2). While it was previously believed that DNA strand breaks were caused directly by high-energy photons, recent evidence suggests that most of the damage is due to oxidative stress [66,67]. In addition to DNA damage, oxidative stress activates redox-sensitive kinases such as Src, PI3K-Akt, and MAPK, including pathways like Erk, JNK, and p38 [68,69,70]. These kinases regulate transcription factors via phosphorylation and have been implicated in cognitive impairment associated with RIBI, especially in the hippocampus’s CA1 region [71,72].

The mitogen-activated protein kinase (MAPK) pathways consist of kinase cascades that regulate key processes such as cell proliferation, differentiation, survival, and apoptosis [73]. Persistent activation of JNK or p38 pathways has been linked to neuronal apoptosis [74]. Ionizing radiation activates all three MAPK pathways, though the intensity of activation depends on cell type [75], with oxidative stress particularly stimulating p38 MAPK [76,77]. Radiation-induced ROS can also activate the ERK cascade, enhancing c-Jun transcriptional activity and upregulating pro-inflammatory genes such as cyclooxygenase-2, interleukin-1β, and tumor necrosis factor-α [78]. Direct exposure of cells to exogenous hydrogen peroxide (H_2_O_2_), mimicking oxidative stress, has similarly been shown to activate MAPK pathways [79,80].

Consequently, the CNS is highly vulnerable to ionizing radiation, highlighting the importance of developing protective therapies [81], with antioxidants emerging as a promising strategy to mitigate such damage [66].

## 4. Hallmarks of Brain Injury Induced by Radiation

Radiation-induced brain injury can manifest with several hallmark features, depending on the stage and severity of the exposure. These effects are typically classified into early (acute and subacute) and late stages; however, these stages can overlap over time.

These effects reflect the cumulative impact of oxidative stress, inflammation, vascular injury, and neural damage triggered by radiation. The severity of these symptoms often depends on the radiation dose, the area of the brain exposed, and the patient’s individual sensitivity to radiation.

### 4.1. Inflammation

This is an immediate response where inflammatory cytokines are released, leading to swelling and disruption of the blood–brain barrier. Current hypotheses suggest that immune cells, particularly the excessive activation of microglia in the CNS and the migration of peripheral immune cells into the brain, play a critical role in initiating and progressing radiation-induced brain injury [82].

Following irradiation, activated microglia release inflammatory factors, exacerbating neuroinflammation and facilitating damage progression. Controlling microglial activation and suppressing the secretion of these factors is therefore crucial for preventing such injuries. While microglial activation is central to neuroinflammation, the precise mechanisms by which radiation triggers this response remain unclear, involving multiple signaling pathways [83,84]. Investigating the interactions among microglia, neurons, astrocytes, and peripheral immune cells may offer strategies to mitigate microglial activation, reduce inflammatory agent release, and limit peripheral immune cell infiltration into the brain.

### 4.2. Brain Edema

After brain trauma, ischemia, inflammation, or the presence of a tumor, water enters astrocytes through AQP4 channels. This leads to brain tissue swelling, cytotoxic edema, and elevated intracranial pressure, all of which can significantly increase morbidity and mortality. Brain edema in radiation-induced brain injury refers to the abnormal accumulation of fluid in the brain tissues as a result of radiation exposure [85]. This swelling occurs because radiation disrupts the blood–brain barrier. Some studies revealed that the water channel protein aquaporin-4 (AQP4) is expressed in perivascular astrocytes end-feet, and regulates water movement across the barrier membrane [86]. In hypoxia- induced cell swelling and damage, AQP4 surface expression increases through a calmodulin-dependent mechanism. The inhibition with trifluoperazine, a typical antipsychotic, reduced AQP4 localization. Therefore, this drug treatment eliminated CNS edema, and promoted faster functional recovery, suggesting that AQP4 inhibition could be a useful therapeutic approach for treating brain edema [87,88]. Indeed, AQP4 trafficking in primary human astrocytes and its vesicular translocation mechanisms are important to the edema treatment [85], and further studies are necessary to determine the effects of this drug on radiation-induced brain injury and in related CNS edema therapies.

In this sense, even the treatment with resveratrol showed that this antioxidant compound ameliorates oxidative stress and inhibits AQP4 in a rat cerebral ischemia-reperfusion injury, being a therapeutic target [89].

### 4.3. Astrogliosis

Astrocytes provide both structural and functional support to neurons and they contribute to angiogenesis, neurogenesis, synaptogenesis, dendrogenesis, and axogenesis, helping to create an environment conducive to recovery. After a brain injury, they suffer a switch in their phenotype into reactive astrocytes and become activated, leading to astrogliosis. This process plays a significant role, acting as both a protective and potentially harmful response in brain injury [90,91].

Astrogliosis involves the proliferation and hypertrophy of astrocytes, which initially help to protect neural tissue by forming a glial scar and preventing further spread of damage, and which have a protective role in recovery from inflammatory and ischemic disease, as well as their role in degenerative disorders [92].

However, chronic or excessive astrogliosis can contribute to long-term detrimental effects. It can exacerbate inflammation, disrupt the blood–brain barrier, and interfere with neuronal function, plasticity and regeneration. Additionally, excessive accumulation of reactive astrocytes can create a non-permissive environment for neural repair, leading to persistent neurological deficits, cognitive impairments, and exacerbation of radiation-induced tissue damage [93,94].

Some studies on strokes showed that the acute inhibition of AQP4 promoted neurological recovery by diminishing brain edema at the early stage and attenuating peri-infarct astrogliosis and AQP4 depolarization at the subacute stage [95]. Indeed, preconditioned extracellular vesicles derived from hypoxic microglia alleviate post-stroke AQP4 depolarization, restore disrupted cerebrospinal fluid flow, and reduce astrogliosis and neuroinflammation [96].

Therefore, due to their dual role in both supporting and hindering recovery, astrocytes have emerged as promising therapeutic targets for pharmacological interventions aimed at improving functional outcomes and neurological recovery.

Additionally, there are other side effects, such as neurological symptoms, including trigeminal nerve deficit, hydrocephalus, ataxia, and dizziness, which may appear in the acute phase [97]. Vascular damage can also occur, as radiation induces fibrosis and damages blood vessels, leading to chronic ischemia and an increased risk of more injury [98,99]. Another serious consequence is radiation necrosis [100], which involves tissue death in localized areas of the brain, often resulting in permanent neurological deficits during the late stage.

## 5. Use of Other Pharmacological Approaches against RIBI in Clinical Setting

In recent years, numerous studies have explored various pharmacological strategies to mitigate neurocognitive toxicity resulting from whole-brain radiotherapy (WBRT). Potential treatments to reduce these hallmarks include memantine, alpha tocopherol, indomethacin, renin–angiotensin system blockers, ACE inhibitors, PPAR-α, melatonin, and metformin [101,102,103]. However, there is a lack of human studies with these compounds and neuroprotective outcomes. An exception is memantine, which can reduce ROS and pyroptosis via NLRP3/NLRC4/Caspase-1 in RIBI [104]. Moreover, a recent study showed that pretreatment with metformin reduces inflammation and decreases DNA damage in the in vitro and downstream pathways involved in RIBI [102].

### 5.1. Memantine

Memantine is a noncompetitive, low-affinity, open-channel antagonist of the N-methyl-D-aspartate receptor (NMDAR). Glutamate serves as the primary excitatory neurotransmitter in cortical and hippocampal neurons, with the NMDAR playing a critical role in learning and memory. WBRT is known to induce profound capillary rarefaction, reduce vascular density, and impair vasculogenesis, all of which contribute to radiation-induced cognitive decline [16].

Memantine has been shown to block ischemia-induced NMDA excitation and has proven effective in treating vascular dementia [105]. As such, it is posited that memantine may provide neuroprotection against radiation-induced cognitive impairment, making it a promising candidate for prophylactic use during radiation therapy [106]. Preclinical models have demonstrated its neuroprotective effects [107,108,109], and in two placebo-controlled Phase III trials, memantine was validated as an effective treatment for small-vessel disease [110,111].

According to Brown et al. (2013), a placebo-controlled Phase III trial involving patients with brain metastases receiving WBRT showed that memantine significantly extended the time to cognitive decline, with neurocognitive function preservation improved by up to 31% in the memantine group [112]. However, it is concerning that nearly 70% of brain-metastases patients treated with WBRT still experienced neurocognitive deterioration within six months, and this figure rose to between 50% and 90% within three to six months following fractionated WBRT [112,113].

In a Phase II multi-institutional clinical trial (RTOG 0933), Gondi et al. (2014) demonstrated that conformal hippocampal avoidance (HA) using intensity-modulated radiation therapy during WBRT is associated with better memory preservation compared to historical control series [13].

Further supporting this, Brown et al. (2020) found in the Phase III clinical trial NRG CC001 that the combination of memantine with HA-WBRT should be considered standard care for patients with a good performance status who are set to undergo WBRT (excluding metastases in the hippocampal region), in order to preserve cognitive function without compromising overall survival or intracranial progression-free survival [114].

Given the persistent rate of neurocognitive decline, additional neuroprotective strategies continue to be investigated to enhance the benefits of memantine and HA protocols [3,112]. A summary of other pharmacological approaches as preclinical interventions for RIBI is presented in Table 1.

### 5.2. Vitamin C or Ascorbic Acid

Vitamin C, also known as ascorbic acid (AA), is one of the most potent antioxidants. It is crucial for the development and maintenance of connective tissues and plays a key role in bone formation, wound healing, and the health of gums. Metabolically, it is involved in several vital processes, including the activation of vitamin B, folic acid, the conversion of cholesterol to bile acids, and the transformation of amino acids, such as tryptophan and serotonin. Its antioxidant properties protect the body from free radical damage, and it has been proposed as a therapeutic agent for various diseases [115].

In particular, AA is an essential micronutrient for the CNS, as will be discussed in the following sections.

#### 5.2.1. Dynamics of Ascorbate in the CNS

Ascorbic acid is highly concentrated in the CNS, particularly in the gray matter, including the hippocampus [116,117]. It is a potent water-soluble antioxidant [107] and an essential micronutrient for the CNS [118,119]. AA enters the CNS via SVCT2 transporters at the choroid plexus, which are stereospecific for the L-isomer [120,121]. From the cerebrospinal fluid (CSF), AA diffuses into the brain’s extracellular fluid, with concentrations ranging from 200 to 400 μM [122,123,124]. In fresh brain tissue, AA concentrations reach 1 to 2.6 mM, approximately one-fifth of glutamate levels [116].

**Figure 3:**
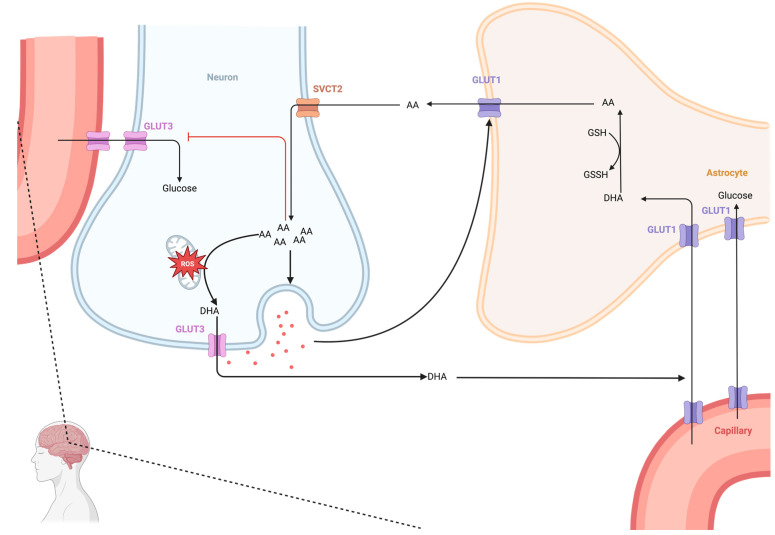
Ascorbate dynamics CNS: uptake and regulation. This figure outlines the distribution and dynamics of ascorbic acid (AA) as it moves from the blood supply into the central nervous system (CNS) to function as an antioxidant. Ascorbate enters the CNS via glucose transporters (GLUTs) as dehydroascorbic acid (DHA) and is reduced to AA in astrocytes. AA is then taken up into neurons from the extracellular fluid (ECF) through sodium-dependent vitamin C transporter 2 (SVCT2), acting as an intracellular antioxidant and oxidizing back to DHA. The recycling of DHA from neurons to the ECF is facilitated by astrocytes. The extracellular concentration of AA is homeostatically regulated and influenced by glutamate release, enhancing transport from astrocytes to the ECF. However, AA recycling may be compromised in pathophysiological conditions. Abbreviations: DHA: Dehydroascorbic acid; GLUT: Glucose transporter; AA: Ascorbic acid; SVCT2: Sodium-ascorbate co-transporters; ROS: reactive oxygen species.

When functioning as an antioxidant, AA is oxidized to dehydroascorbic acid (DHA) [125]. DHA crosses the blood–brain barrier via GLUT1 transporters [126]. Cells subjected to oxidative stress, such as epithelial cells, microglia, and stromal cells, produce superoxide via NADPH oxidase, which oxidizes AA to DHA, facilitating its transport [115]. DHA is then taken up by astrocytes through GLUT1 and reduced back to AA, preventing the loss of oxidized AA [115] (Figure 3).

This recycling of AA is facilitated by enzymes such as semidehydroascorbate reductase, which converts the ascorbyl radical back to ascorbate, and dehydroascorbate reductase, which performs the same conversion on DHA [127,128]. These mechanisms are highly active in brain regions rich in ascorbate, ensuring its recirculation [117] (Figure 3).

Astrocytes, with their higher glutathione concentrations, are primarily responsible for reducing DHA to AA. AA is then released into the interstitial space, providing extracellular antioxidant protection, a process stimulated by glutamate release [126,129]. Some AA is taken up by neurons via SVCT2 transporters, delivering intracellular protection [130].

Within neurons, AA can inhibit glucose consumption and stimulate lactate transport. It also accumulates in mitochondria, where it protects mitochondrial membranes and DNA from free radicals [122,131]. The high concentrations of AA in brain tissue and CSF underscore its vital role in maintaining CNS homeostasis. AA has been shown to modulate both glutamate- and dopamine-mediated neurotransmission [104] and promote myelin formation by Schwann cells, while scavenging free radicals [130,132].

#### 5.2.2. Antioxidant Effects of Ascorbic Acid

**Figure 4:**
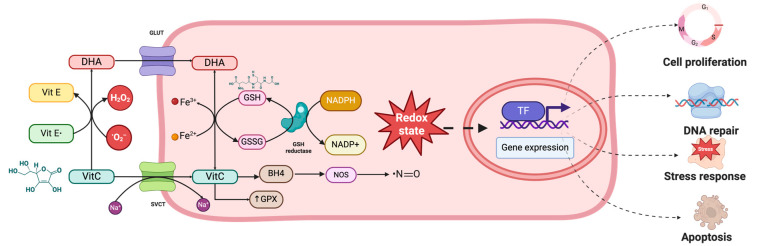
Mechanism of AA uptake and its effects in the cell. Studies with cultured cells have shown that AA can affect gene expression mediated by its redox effects. Abbreviations: DHA, dehydroascorbic acid; Fe^3+^, ferric iron; Fe^2+^, ferrous iron; GPX, glutathione peroxidase; GSH, reduced glutathione; GSSG, oxidized glutathione; GST, glutathione transferase; Vit C, vitamin C; Vit E, vitamin E.

Ascorbic acid (AA) is likely the most important water-soluble antioxidant in the brain’s extracellular fluid. It plays a key role in reducing excitotoxicity [133] and is essential for regenerating reduced α-tocopherol in cell membranes [134], thereby protecting lipids, proteins, and DNA from oxidative damage and maintaining their normal structure and biological function (Figure 4).

AA halts oxidative stress by donating a hydrogen atom to reactive oxygen species (ROS), leading to the formation of a stable ascorbyl radical—a direct antioxidant mechanism. This action provides protection against oxygen-derived molecular species [135]. Oxidative damage to biomolecules produces measurable by-products, such as 8-oxodeoxyguanosine from DNA [136], F2-isoprostanes from lipids [137] and carbonyl derivatives from proteins [138]. These markers offer valuable methods to assess AA’s antioxidant effects. Additionally, the ascorbyl radical can be monitored using electron paramagnetic resonance spectroscopy to evaluate oxidative stress, as has been carried out in patients with sepsis [139,140].

Due to its potent antioxidant properties, AA is considered an important neuroprotective agent [141]. However, while AA counteracts oxidative stress, it can also form reactive oxidants, particularly in the presence of transition metals [142]. Furthermore, AA exerts indirect antioxidant effects by inhibiting ROS-producing enzymes and reducing inflammatory responses via NF-κB pathway inhibition [66] (Figure 3).

### 5.3. The Role of Ascorbic Acid in Preventing Radiation-Induced Brain Injury (RIBI)

The protective role of AA in preventing RIBI has garnered significant attention in recent studies. AA exhibits potent antioxidative properties that can mitigate the cellular damage caused by ionizing radiation. Research indicates that the lipophilic vitamin C derivative, 6-o-palmitoylascorbate, shows superior efficacy compared to regular ascorbate in reducing X-ray-induced DNA damage, lipid peroxidation, and protein carbonylation in human lymphocytes [143]. This derivative not only enhances cell viability but also prevents the depletion of crucial antioxidants like glutathione, thus offering substantial protection against oxidative stress induced by radiation.

In addition to its protective effects, AA has been shown to significantly reduce DNA double-strand breaks (DSBs) associated with radiation exposure. A randomized, double-blind, placebo-controlled trial found that pre-treatment with AA led to an impressive 87% reduction in DSBs in patients exposed to high radiation doses during cardiac examinations [144]. Moreover, oral administration of AA prior to abdominal contrast-enhanced CT scans resulted in a 61% reduction in the mean increase of γ-H2AX foci, further underscoring its capacity to protect against genetic damage from radiation [145].

The cumulative evidence suggests that AA, especially when used in conjunction with other antioxidants like N-acetylcysteine, may play a crucial role in enhancing cellular defenses against RIBI. Its ability to scavenge free radicals and stabilize genomic integrity highlights its potential as a therapeutic agent in clinical settings involving radiation exposure [146]. Thus, incorporating vitamin C into pre-radiation protocols could be a valuable strategy for reducing the risk of RIBI and improving patient outcomes.

### 5.4. The Dual Effect of Ascorbic Acid in Cancer

Ascorbic acid (AA) has been shown to enhance tumor radiosensitization while simultaneously reducing radiation-induced toxicity in normal tissues, particularly in non-CNS models of pancreatic cancer [147]. However, the overall role of antioxidants in cancer remains a topic of considerable debate. On one hand, antioxidants serve a protective function by neutralizing free radicals, thereby reducing oxidative stress and potentially lowering the risk of cancer development by preventing DNA damage. This protective mechanism is crucial, as oxidative stress is a well-established factor in the initiation of cancer [148,149].

Conversely, once cancer has developed, the effects of antioxidants can become paradoxical [150,151]. High doses of AA have been reported to exhibit various antitumor effects, including the proteolysis of hypoxia-inducible factor alpha (HIFα), epigenetic regulation, and a pro-oxidant effect. This oxidative-promoting action at elevated concentrations can be detrimental to cancer cells, as noted by Cockfield and Schafer [152], who discuss the context-specific vulnerabilities of cancer cells to antioxidant defenses. Furthermore, by reducing oxidative stress, antioxidants may inadvertently protect cancer cells from damage caused by reactive oxygen species, which could otherwise be therapeutically employed to induce cancer cell death. This protective mechanism may facilitate tumor progression and confer resistance to certain cancer treatments. Thus, while antioxidants are beneficial in the prevention of cancer, their role in established cancers is complex and may vary based on the context and specific cancer type, necessitating further investigation beyond just brain cancers.

Interestingly, Levine et al. highlight that AA can deliver hydrogen peroxide (H_2_O_2_) to tumor cells and participate in Fenton reactions involving redox-active intracellular iron, leading to oxidative damage [153]. In glioblastoma, a Phase 2 clinical trial involving pharmacologic ascorbate combined with chemoradiation demonstrated a significant increase in median overall survival, rising from 14.6 months in historical controls to 19.6 months in the trial cohort [154]. This evidence underscores the nuanced role of vitamin C in cancer therapy and the need for tailored approaches in its application.

**Table 1:** Studies accounting for pharmacological strategies against RIBI.

Details of the Study	Model	Groups	Irradiation Procedure	Drug Tested	Cognitive Testing	Other Evaluations	Results	Ref.
MitoQ	KM	Divided into four groups, 10 mice each: 1. G1: ip PSS 0.9% for 3 days 2. G2: ip MitoQ 3. G3: WBI 4. G4: ip MitoQ + WBI	WBI of mice was performed using a high-LET 56-Fe ions beams at the energy of 160 MeV/μ. Each mouse received 2 Gy doses at a dose rate of 0.5 Gy/min, and the mice were placed in the plateau region.	MitoQ groups received MitoQ (5 mg/kg/day) for 3 days	(-)	-Determination of oxidative stress parameters (PCO, MDA, SOD, CAT) -Mitochondrial respiration measurements (O2 consumption and RCR) -Measurement of mitochondria-generated ROS -mtDNA damage assay -mitochondrial dynamics protein (Mfn2, Drp1, bcl-2, bax, cyto c) -Gene expression analysis (BA; Casp3; SOD2; Opa1)	MitoQ reduced radiation-induced oxidative stress with decreased lipid peroxidation and reduced protein and DNA oxidation. MitoQ protected mitochondrial respiration after RT. MitoQ increased Mfn2 and OPA1 and decreased Drp1. MitoQ also suppressed mitochondrial DNA damage, cyto c release, and caspase-3 activity in RT-treated mice compared to the control group [152].	[155]
Quercetin	WAR	Divided into 4 groups (n = 8/each): 1. control group 2. G QUER: quercetin 3. G RAD was given only irradiation 4. G RAD + QUER: quercetin + irradiation	RAD groups were subjected to cranium irradiation with a single dose of 20 Gy of photons using a 6 MV LINAC at a dose rate of ~1 Gy/min, with the source–axis distance technique, with 1.0 cm of bolus material on the surface.	QUER groups received Quercetin 50 mg/kg body weight (BW) daily in distilled water and 0.25 mL PS for 15 days.	(-)	-Total antioxidant status and MDA -Brain histopathological evaluation	Tissue samples and biochemical levels of tissue-injury markers in the four groups were compared. In all measured parameters of oxidative stress, administration of quercetin significantly demonstrated favorable effects. Both plasma and tissue levels of MDA and total antioxidant status significantly changed in favor of antioxidant activity. Histopathological evaluation of the tissues also demonstrated a significant decrease in cellular degeneration and infiltration parameters after quercetin administration. Quercetin demonstrated significant neuroprotection after radiation-induced brain injury.	[156]
Date syrup	WAR	Divided into 4 groups, 15 rats each. 1. G1 (Control); received 1 mL 0.9% saline solution orally for 4 weeks and served as control; 2. G2 (Irradiated); was exposed to radiation at a dose level of 6 Gy and sacrificed after 48 h. 3. G3 (Date syrup); 4. G4 (Irradiated + Date syrup)	Whole-body gamma-irradiation. Animals were irradiated at an acute Single-dose level of 6 Gy delivered at a dose rate of 0.713 rad/s.	Date syrup group received daily date syrup by stomach intubation at a dose of 4 mL/kg body weight for 4 weeks.	(-)	-Serum biochemical analysis. -Assessment of oxidant/antioxidant biomarkers (lipid peroxidation, DNA damage, GSH, CAT activity -Assessment of MMP-9 -q RT-PCR evaluation for TNF-α gene expression -Liver histopathological examination	Pretreatment of rats with Date syrup ameliorated the tissue damage induced by radiation as evidenced by the improvement in liver function, antioxidant status and reduction in DNA damage. Moreover, liver TNF-α expression and serum MMP-9 activity were reduced.	[157]
NSI-189	LER	Divided into 3 groups (n = 15–16/each): 1. controls receiving oral gavage (vehicle only) and sham irradiation 2. cohorts receiving oral gavage (vehicle only) and 27 Gy head-only fractionated exposure 3. cohorts receiving oral gavage (NSI-189, 30 mg/kg) and 27 Gy head-only fractionated exposure	For CI, animals were positioned under a collimated (1 cm^2^ diameter) beam for head-only irradiation delivered at a dose rate of 1 Gy/min. Fractionation of 27 Gy was delivered over 3 separate doses of 8.67 Gy, which were administered 48 h apart.	NSI 189 The drug was administered by daily oral gavage at a concentration adjusted to the weight of the animals. The daily dosing was set at 2 mL/kg, setting the target daily dose of 30 mg/kg. Thus, the daily volume of the drug typically varied between 0.6 and 1.0 mL/rat.	cognitive testing 1 week after termination of oral gavage (5 weeks post-RT). Cognitive testing was performed over the course of three weeks and included four different spontaneous exploration tasks (novel place recognition, novel object recognition, object in place and temporal order) followed by contextual and cued fear conditioning	-Assessment of neurogenesis -Determination of hippocampal volume -Assessment of activated microglia	NSI-189 treatment resulted in significantly improved performance in four of these tasks: novel-place recognition, novel-object recognition, object in place and temporal order. In addition, there was a trend for improved performance in the contextual phase of the fear-conditioning task. Importantly, enhanced cognition in the NSI-189-treated cohort was found to persist one month after the cessation of drug treatment. These neurocognitive benefits of NSI-189 coincided with a significant increase in neurogenesis and a significant decrease in the numbers of activated microglia compared to the irradiated cohort that was given the vehicle alone.	[158]
Fingolimod	CM	Divided into 4 groups: 1. G1: methylcellulose vehicle alone 2. G2: vehicle + radiation 3. G3: FTY720 4. G4: FTY720 + radiation	For irradiation, a Gammacell 40 irradiator with a dose rate of 95. cGy/minute was used. A single dose of 7 Gy was administered to each animal.	FTY720 groups received three ip of 0.5 mg/kg FTY720 in the week prior to irradiation. They then received 3 ip/week of vehicle or 0.5 mg/kg FTY720 for 6 weeks.	Fear conditioning and MWM were then employed to test learning and memory.	-IF and IHC of brain tissue (antibodies: anti S1PR1, nestin, GFAP, doublecortin, NeuN, Tubulin III/Tuj1) -qRT-PCR of BDNF vs. B2	The learning deficits were fully restored by FTY720. In irradiated brains, FTY720 maintained the cytoarchitecture of the dentate gyrus granular cell layer and partially restored the pool of NPC. In mice harboring BTSC xenografts, FTY720 delayed tumor growth and improved survival.	[159]
mNGF	SDR	Divided into 3 groups: G1: control (n = 15) G2: mNGF + CI (n = 20) G3: PSS + CI (n = 20)	CI at a single dose of 12 Gy by X-ray.	?	MWM experiment	EB leakage of the brain, and expressions of neuN, vWF, ZO-1 in hippocampus by immunofluorescence, and expressions of neuN, vWF, ZO-1, VEGF and GFAP in hippocampus by WB	mNGF decreases the damage by RT, improving the latency time of escape in the Morris water maze, and decreases the EB leakage. In the IF, mNGF increases the expression of neunN, vWF abd ZO-1. In WB, mNGF increases the expression of neuN, vWF and ZO-1.	[160]
Kukoamine (KuA)	WAR	Divided into 5 groups (n = 5–8/group): 1. G1: sham irradiation 2. G2: CI 3. G3: CI + KuA low dose 4. G4: CI + KuA middle dose 5. G5: CI + KuA high dose	CI was performed with 6-MeV electron beams delivered by a LINAC. Irradiated rats received a single dose of 30 Gy X-rays at a dose rate of 250 cGy/min.	KuA was administered at a dose of 5, (G3) 10 (G4) and 20 mg/kg (G5) body weight.	(-)	-MDA, GSH level and SOD, CAT activity assays -Nissl Staining and TUNEL staining -WB, using antibodies anti: BDNF, Casp3, CytC, Bax, Bcl2, GAPDH, BA	Whole brain irradiation led to the neuronal abnormality and it was alleviated by KuA. KuA decreased MDA level, increased GSH level, SOD and CAT activities, as well as alleviated neuronal apoptosis by regulating the expression of cleaved caspase-3, cytochrome C, Bax and Bcl2. Additionally, KuA increased the expression of BDNF.	[15]
Acanthopanax	KM	Divided into 3 large groups (n = 32 each) G1: behavioral test G2: pathological sections G3: metabolomics analysis. Each large group was divided into 4 small groups for the experiments (n = 8 per group) g1: normal control g2: model set (CI) g3: treatment group AS + CI g4: treatment group V + CI	Irradiated by 60 Co-γ ray irradiation with the mean LET of 62.2 KeV/μm at a dose of 4 Gy and a dose rate of 0.1 Gy/min.	AS was administered at a dose of 235.7 mg/kg/day. V was administered at a dose of 13.75 mg/kg/day.	MWM and sucrose preference test	-Production of pathological sections for brain tissues (PFC) -Metabolomics analysis based on 1H NMR	AS significantly improved the decline of low LET-induced learning ability and spatial memory capacity, increased the sensitivity of the nervous system and, to a certain degree, prevented brain tissue lesions caused by radiation. In our study, we also observed that AS had a better effect on brain tissue development and brain–glutamate-cycle balance compared with a chemical drug (Venlafaxine).	[161]

NOTES: WAR: Wistar albino rats; LER: Long–Evans rats; KM: Kunming mice; MDA: malondialdehyde; WBI: whole body irradiation; LINAC: linear accelerator; ip: intraperitoneal injection; PSS: physiological saline solution; G: group; PCO: protein carbonyl; SOD: superoxide dismutase; CAT: catalase; RCR: respiratory control ratio; CM: C57/Bl/6J mice; BTSC: brain tumor stem cell; IF: Immunofluorescence; IHC: immunohistochemistry; SDR: Sprague Dawley rat; CI: cranial irradiation; EB: Evans blue; WB: Western blot; mNGF: mouse nerve growth factor; BA: beta-actin; MWM: Morris water maze; V: venlafaxine; AS: acanthopanax senticosus; PFC: prefrontal cortex.

## 6. Discussion and Conclusions

In conclusion, emerging evidence highlights the protective role of AA and other compounds such as memantine in mitigating RIBI. AA’s potent antioxidative properties can significantly reduce cellular damage caused by ionizing radiation. Notably, the lipophilic derivative 6-o-palmitoylascorbate has shown greater efficacy than standard ascorbate in minimizing X-ray-induced DNA damage, lipid peroxidation, and protein carbonylation in human lymphocytes. This derivative enhances cell viability and preserves essential antioxidants like glutathione, providing substantial protection against oxidative stress from radiation exposure.

Moreover, clinical studies demonstrate AA’s ability to substantially decrease DNA double-strand breaks associated with radiation. For instance, a randomized trial revealed an impressive 87% reduction in double-strand breaks in patients pre-treated with AA before high-dose radiation procedures. Similar results were observed with oral AA administration prior to CT scans, resulting in a 61% decrease in the mean increase of γ-H2AX foci.

While the role of MAPK-mediated signaling in RIBI is under investigation, the specific impact of antioxidants like AA and other drugs remains largely unexplored. Concerns about potential detrimental effects in anticancer therapies exist; however, recent research indicates that AA does not compromise the efficacy of radiotherapy. There is an urgent need for comprehensive studies to elucidate the relationship between AA and standard pharmacological treatments in clinical settings aimed at preventing RIBI.

Incorporating AA or memantine into pre-radiation protocols could prove invaluable for reducing RIBI risk and enhancing patient outcomes. Future clinical trials should focus on evidence-based assessments, including neurocognitive tests, to evaluate the effectiveness of antioxidant interventions and develop more effective strategies for managing RIBI.

## Data Availability

No datasets were generated or analyzed during the current study.

## Abbreviations

AA	ascorbic acid
AQP4	aquaporin-4
BMs	brain metastases
CAT	catalase
CD	cell death
CNS	central nervous system
CSF	cerebrospinal fluid
DDR	DNA damage response
DHA	dehydroascorbic acid
DSB	double-strand break
ERK	extracellular-signal-regulated kinase
FRs	free radicals
GPx	glutathione peroxidase
GSH	reduced glutathione
HR	homologous recombination
IAP	intrinsic apoptotic pathway
JNK	c-Jun N-terminal kinase
LQ	linear quadratic
MAPK	mitogen-activated protein kinase
NHJE	non-homologous end-joining
NMDAR	N-methyl-D-aspartate receptor
RIBI	radiation-induced brain injury
ROS	reactive oxygen species
RT	radiotherapy
SOD	superoxide dismutase
SSB	single-strand break
TME	tumor microenvironment
WBRT	whole brain radiation therapy
